# Preparation of Sandwich-like NiCo_2_O_4_/rGO/NiO Heterostructure on Nickel Foam for High-Performance Supercapacitor Electrodes

**DOI:** 10.1007/s40820-016-0117-1

**Published:** 2016-11-28

**Authors:** Delong Li, Youning Gong, Miaosheng Wang, Chunxu Pan

**Affiliations:** 1 Shenzhen Research Institute, Wuhan University, Shenzhen, 518057 People’s Republic of China; 2 School of Physics and Technology, Center for Electron Microscopy and MOE Key Laboratory of Artificial Micro- and Nano-structures, Wuhan University, Wuhan, 430072 People’s Republic of China; 3 Department of Materials Science and Engineering, University of Tennessee, Knoxville, TN USA

**Keywords:** NiCo_2_O_4_, Reduced graphene oxide (rGO), NiO, Heterostructure, Supercapacitors

## Abstract

**Abstract:**

A kind of sandwich-like NiCo_2_O_4_/rGO/NiO heterostructure composite has been successfully anchored on nickel foam substrate via a three-step hydrothermal method with successive annealing treatment. The smart combination of NiCo_2_O_4_, reduced graphene oxide (rGO), and NiO nanostructure in the sandwich-like nano architecture shows a promising synergistic effect for supercapacitors with greatly enhanced electrochemical performance. For serving as supercapacitor electrode, the NiCo_2_O_4_/rGO/NiO heterostructure materials exhibit remarkable specific capacitance of 2644 mF cm^−2^ at current density of 1 mA cm^−2^, and excellent capacitance retentions of 97.5% after 3000 cycles. It is expected that the present heterostructure will be a promising electrode material for high-performance supercapacitors.

**Graphical Abstract:**

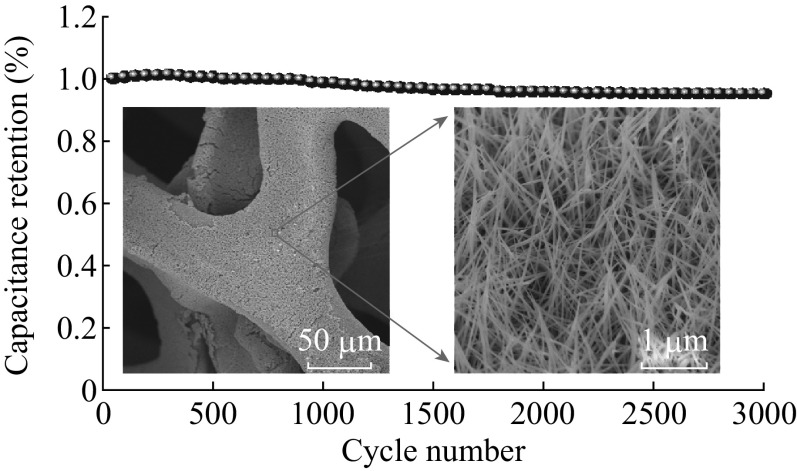

## Highlights

- A new sandwich-like NiCo_2_O_4_/rGO/NiO heterostructure is prepared by using a facile process via a three-step hydrothermal method.
- This sandwich-like heterostructure exhibits a specific capacitance up to 2644 mF cm^−2^ and shows enhanced electrochemical performance.

## Introduction

Supercapacitors have attracted wide attention because of their ultra-high power density, long cycling stability, fast charge/discharge rate, and bridging function for the power and energy gaps between batteries and traditional dielectric capacitors [1, 2]. Generally, the electrode materials used for supercapacitors can be divided into two categories based on the different energy storage mechanisms: electrical double-layer capacitors (EDLCs) and pseudo-capacitors (PCs) [3]. In fact, PCs exhibit much larger capacitance values and energy density than EDLCs due to their fast and reversible redox reaction [4]. Therefore, considerable efforts have been focused on improving the performance of PCs. It has been recognized that the most used PCs’ electrode materials, including RuO_2_, NiO, Co_3_O_4_, and MnO_2_, possess multiple oxidation states/structures, which enable rich redox reactions on the surface of the electrodes and provide high specific capacitance. However, the poor conductivity and cycling stability of these materials restrict their applications [5–8].

More recently, mixed transition-metal oxides (MTMOs), such as single-phase ternary metal oxides with two different metal cations, typically in a spinel structure (donated as A_x_B_3−x_O_4_, A, B = Co, Ni, Zn, Mn, Fe, and so on), have captured much attention as promising electrode materials in electrochemical energy conversion and storage systems [9, 10]. Among the MTMOs, compared with NiO and Co_3_O_4_, the spinel nickel cobaltite (NiCo_2_O_4_) exhibits better electrical conductivity and higher electrochemical activity [9, 11]. However, the relatively weak conductivity and small specific surface area make the capacity greatly lower than the theoretical value. Therefore, numerous efforts have been made to optimize the supercapacitors performance of NiCo_2_O_4_ via various methods, including control of microstructures, crystallinity, and electrical conductivity [12–14].

Rationally designed electrode materials with well-defined micro-/nanostructures are attractive methods to enhance the performance of PCs [15–18]. For example, Zhang et al. [15] reported Co_3_O_4_@NiCo_2_O_4_ nanowire arrays for PCs with an improved specific capacitance (2.04 F cm^−2^ at 5 mV s^−1^) with respect to pure Co_3_O_4_. Liu et al. [16] synthesized a binder-free hierarchical NiCo_2_O_4_/NiO nanowire array using a facile hydrothermal method, and the composites exhibited superior pseudocapacitive performance with high specific capacitance (2220 F g^−1^ at 1 A g^−1^). Cai et al. [17] constructed a one-dimension (1D) CNT@NiCo_2_O_4_ core–shell structural nanocable for PCs with a high capacitance of 1038 F g^−1^ at 0.5 A g^−1^.

In addition, graphene has been widely used as electrode materials due to its excellent electrical, optical, and chemical properties [19–22]. Zhang et al. [20] showed that flower-like NiCo_2_O_4_/3D graphene foam exhibited a specific capacitance of 1402 F g^−1^ at 1 A g^−1^. Liu et al. [21] prepared a mesoporous NiCo_2_O_4_ nanoneedle grown on graphene networks and the composite delivered a high specific capacitance of 970 F g^−1^ at 20 A g^−1^.

In this paper, we prepared a sandwich-like NiCo_2_O_4_/rGO/NiO heterostructure composite on nickel foam (NF) via a facile hydrothermal method and subsequent annealing in the air. In the designed sandwich-like structure, the components were assembled into a uniform structure and each component could partially retain its individual traits to improve electrochemical properties. When it was used as electrode materials, it showed a much higher specific capacitance than those of materials such as NiCo_2_O_4_, NiO, and NiCo_2_O_4_/NiO. We propose that the smart combination among NiO, rGO, and NiCo_2_O_4_ nanostructures may provide a synergistic effect for supercapacitors to enhance the electrochemical performance.

## Experimental Section

### Preparation of NiO on NF

Prior to the synthesis, the NF substrate was carefully cleaned with acetone, ethanol, and deionized (DI) water in an ultrasound bath to remove surface impurities. In a typical procedure, the cleaned NF (approximately 1 × 1 cm^2^ for each piece) and 40 mL DI water were put into a 50-mL Teflon-lined stainless autoclave and heated at 200 °C for 24 h. The precursor products were washed with ethanol and DI water, and dried at 80 °C for 6 h. Then, the products were annealed at 350 °C for 2 h in air to obtain NiO on NF. The load mass of NiO is 0.6 mg cm^−2^.

### Preparation of rGO/NiO Composite on NF

Graphene oxide (GO) was synthesized by a modified Hummer’s method which is described in detail in our previous work [22]. The rGO/NiO composites were prepared according to the following process. (i) The as-synthesized GO (40 mg) was added into DI water (40 mL) and dispersed for 2 h with an aid of ultra-sonication. (ii) Several pieces of NiO on NF were put into the above GO dispersion, followed by soaking for 2 h. (iii) The mixture was then transferred into a 50 mL Teflon-lined stainless autoclave, and maintained at 200 °C for 24 h. (iv) As the autoclave cooled down to room temperature, the products were washed for several times with DI water and ethanol, and dried at 60 °C for 4 h in vacuum to obtain the rGO/NiO composites on NF. The load mass of rGO/NiO was 0.6 mg cm^−2^ (the mass of rGO was too small to be weighed out).

### Preparation of Sandwich-like NiCo_2_O_4_/rGO/NiO Heterostructure Composites on NF

The typical synthesis process of sandwich-like NiCo_2_O_4_/rGO/NiO heterostructure composites on NF is as follows. Firstly, 0.5 g urea, 1 mmol Co(NO_3_)_2_·6H_2_O, and 0.5 mmol Ni(NO_3_)_2_·6H_2_O were dissolved into 40 mL DI water, and stirred for 30 min to form a uniform solution. Then, a piece of rGO/NiO on NF was put into the above solution and soaked for 2 h. The mixture was transferred into a 50 mL Teflon-lined stainless autoclave, and maintained at 120 °C for 6 h. As the autoclave cooled down to room temperature, the precursor products were washed for several times with DI water and ethanol, and dried at 60 °C for 4 h under vacuum. Finally, the precursors were annealed at 350 °C for 2 h to obtain sandwich-like NiCo_2_O_4_/rGO/NiO heterostructure on NF. The NiCo_2_O_4_ or the NiCo_2_O_4_/NiO composite on NF were fabricated under identical conditions, in which the substrate was changed into NF or NiO on NF. The load mass of the NiCo_2_O_4_/rGO/NiO, NiCo_2_O_4_/NiO, and NiCo_2_O_4_ are 1.6, 1.4, and 0.9 mg cm^−2^, respectively.

### Materials Characterizations

The phase structures of the samples were characterized by using an X-ray diffraction spectrometer (XRD, D8 Advanced XRD; Bruker AXS, Karlsruhe, Germany) with Cu *K*α radiation. The morphologies of the samples were observed by using a scanning electron microscope (SEM, S-4800, Hitachi High-Technologies Corporation, Japan). Raman spectra were measured in a laser scanning confocal micro-Raman spectrometer (LabRAM HR, HORIBA, France).

### Electrochemical Measurement

The electrochemical tests were performed in a 6 M KOH aqueous electrolyte solution at room temperature. The electrochemical properties of the samples were evaluated using a CHI660D Electrochemical Working Station in a three-electrode system, wherein the samples on NF function as the working electrode (WE), platinum functions as the counter electrode, and saturated calomel electrode (SCE) electrode functions as the reference electrode.

The specific capacitance (*C*) was calculated from the slope of each discharge curve, according to the equation $C = (I \times \Delta t)/(\Delta V \times S),$ where *I* is the constant discharge current; Δ*t* is the discharge time; Δ*V* is the voltage difference in discharge (exclude IR drop); and *S* is the area of each active materials [19, 23]. Electrochemical impedance spectroscopy (EIS) measurements were made in the frequency range of 0.1–100,000 Hz by applying an AC voltage with 5 mV perturbation.

## Results and Discussion

**Fig. 1:**
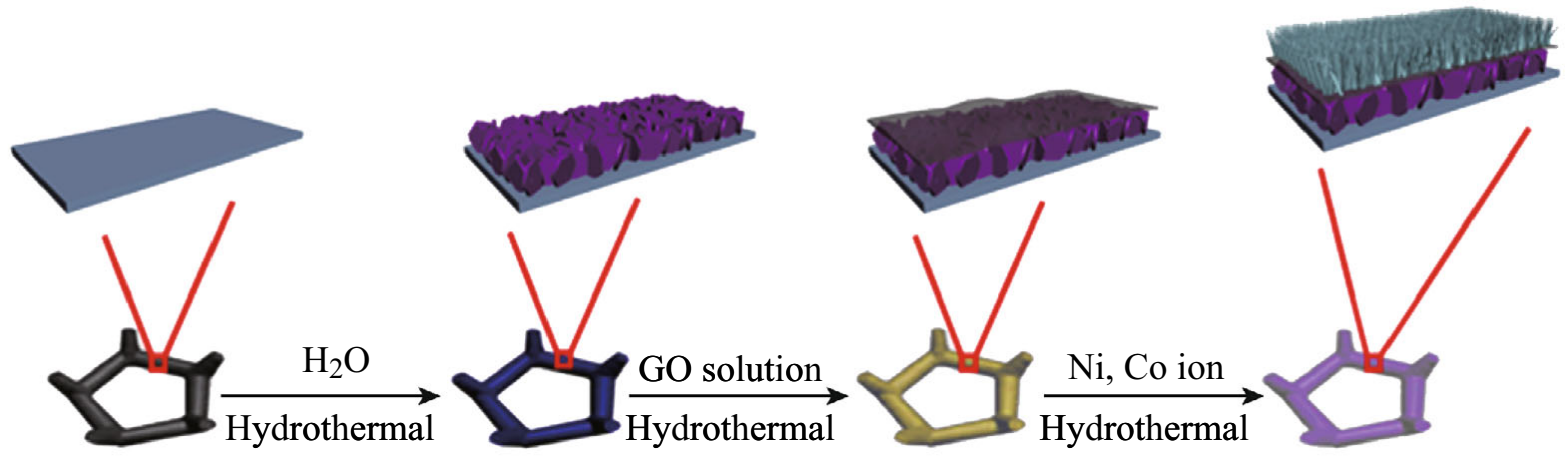
Schematic illustration of the preparation process of the sandwich-like NiCo_2_O_4_/rGO/NiO heterostructure

The general preparation process and the resulting novel supercapacitors electrode heterostructure materials are schematically illustrated in Fig. 1. A multi-step hydrothermal method followed by a calcination process was employed to prepare the sandwich-like NiCo_2_O_4_/rGO/NiO heterostructure composite.

**Fig. 2:**
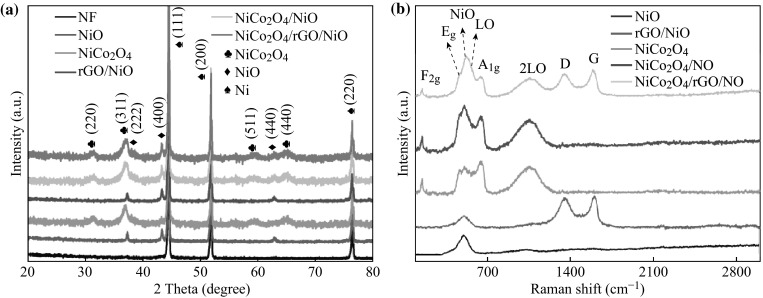
**a** XRD patterns, and **b** Raman spectra of the NF, NiO, rGO/NiO, NiCo_2_O_4_, NiCo_2_O_4_/NiO, and NiCo_2_O_4_/rGO/NiO. (Color figure online)

The XRD patterns of as-prepared samples are shown in Fig. 2a, in which the samples are NF, NiO, rGO/NiO, NiCo_2_O_4_, NiCo_2_O_4_/NiO, and NiCo_2_O_4/_rGO/NiO, respectively. As the active materials directly grew on the surface of NF, the strong typical peaks were ascribed to NF substrate. The diffraction peaks of NiCo_2_O_4_ and NiO were also observed clearly. However, the diffraction peak of rGO was not identified due to the low mass content of rGO. In the Raman spectra of the samples as shown in Fig. 2b, the peaks of rGO were clearly observed at 1349.8 and 1590.2 cm^−1^, which are corresponding to the D and G band of rGO. The peaks located at 151.6, 457.6, 455.2, 505.7, 656.6, and 1096.4 cm^−1^, respectively, correspond to F_2g_, E_g_, L_O_, A_1g_, and 2 L_O_ modes of NiCo_2_O_4_, while the peak located at 501.3 cm^−1^ belongs to NiO. These results are well consistent with the previously reported literatures [18, 21, 24].

**Fig. 3:**
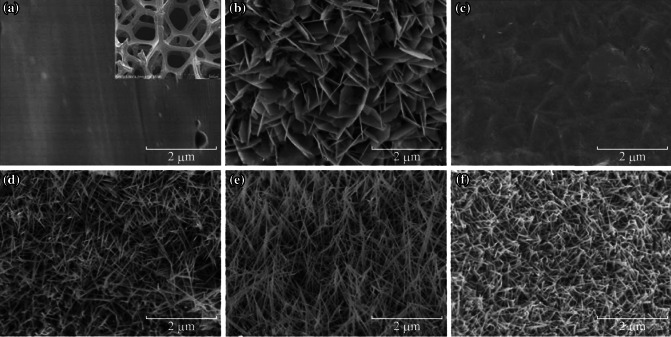
SEM morphology of the samples: **a** NF, **b**NiO, **c** G/NiO, **d** NiCo_2_O_4_, **e** NiCo_2_O_4_/NiO, and **f** NiCo_2_O_4_/rGO/NiO

**Fig. 4:**
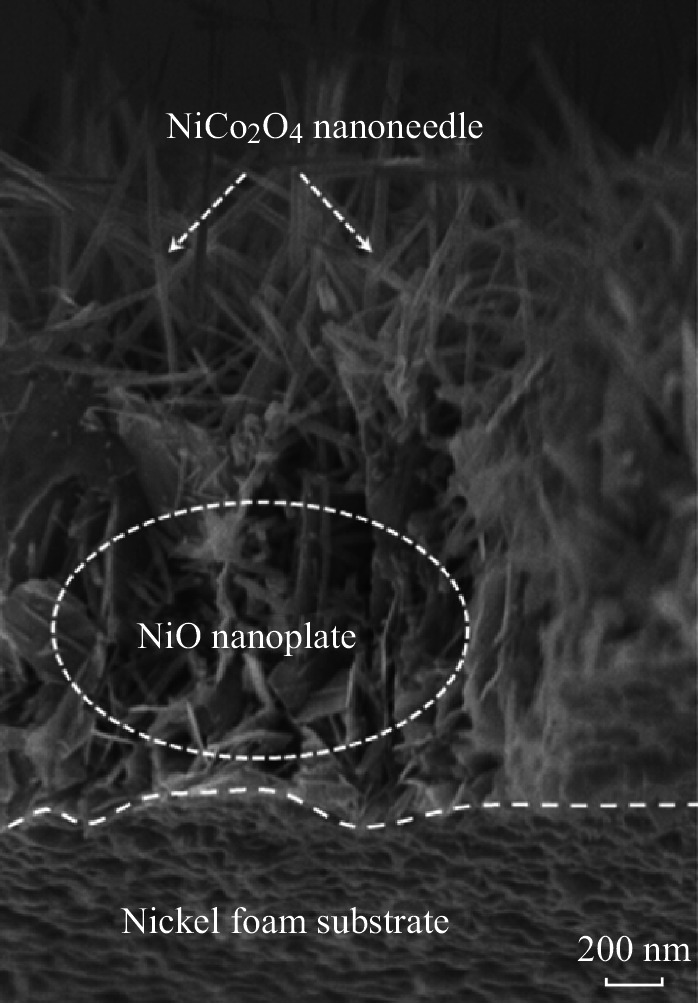
SEM cross-section view of NiCo_2_O_4_/rGO/NiO

Figure 3 shows the top-view SEM images of the samples of NF, NiO, rGO/NiO, NiCo_2_O_4_, NiCo_2_O_4/_NiO, and NiCo_2_O_4_/rGO/NiO. Comparing with original NF, the NF surface is covered with the NiO nanoplates after the first hydrothermal treatment (see Fig. 3b). And then an rGO layer was observed on the surface of the NiO nanoplates after the second hydrothermal treatment, as shown in Fig. 3c. Figure 3d–f indicates that NiCo_2_O_4_ nanoneedles are directly grown on the surfaces of the NF, NiO, and rGO/NiO, respectively. Obviously, the morphology of NiCo_2_O_4_ is not affected by the substrates. The cross-section SEM image of the sandwich-like NiCo_2_O_4_/rGO/NiO heterostructure composite is shown in Fig. 4, in which the NiCo_2_O_4_ nanoneedle, NiO nanoplate, and NF substrate appear obviously. The rGO thin film was too thin to be observed here.

**Fig. 5:**
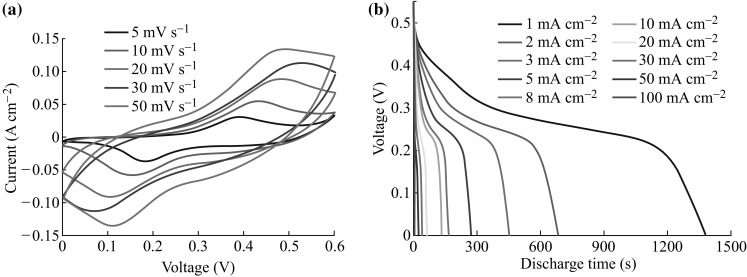
Electrochemical properties of the sandwich-like NiCo_2_O_4_/rGO/NiO heterostructure: **a** CV curves with scan rate change, and **b** GCD curves with current density change. (Color figure online)

The electrochemical properties of the samples were measured by various techniques involving cyclic voltammetry (CV), galvanostatic charge/discharge (GCD), and EIS in a three-electrode system. Figure 5a illustrates the CV curves of the sandwich-like NiCo_2_O_4_/rGO/NiO heterostructure electrode at various scan rates of 1–10 mV s^−1^ in the potential range of 0–0.6 V versus SCE. A couple pair of redox peaks was observed in the CV curves, indicating that the measured capacitance was mainly based on the redox mechanism [25].

In fact, during the charging–discharging process, there exist valence state changes of Co^3+^/Co^4+^, as well as M^2+^/M^3+^ (M = Co or Ni) on the surface of the electrode materials, where fast and reversible Faradaic reactions occur. The Faradaic reactions of NiCo_2_O_4_ in the alkaline electrolyte would proceed according to the following reaction equations [25, 26],

1 $${\text{NiCo}}_{2} {\text{O}}_{4} + {\text{ OH}}^{ - } \leftrightarrow {\text{ NiOOH }} + \, 2{\text{CoOOH }} + 2{\text{e}}^{ - }$$

2 $${\text{CoOOH }} + {\text{ OH}}^{ - } \leftrightarrow {\text{ CoO}}_{2} + {\text{ H}}_{2} {\text{O }} + {\text{ e}}^{ - } .$$

For NiO, the surface Faradaic reactions in the alkaline electrolyte will proceed according to the following equations [27, 28],

3 $${\text{NiO }} + {\text{ OH}}^{ - } \leftrightarrow {\text{ NiOOH }} + {\text{ e}}^{ - }$$

However, the electrochemical redox potentials of the M^2+^/M^3+^ and Co^3+^/Co^4+^ transitions are so close that the observed redox peaks are overlapped [26, 27].

With the increasing scan rate, the redox peaks maintain stable, indicating excellent kinetic reversibility at a large scan rate. To further calculate the specific capacitance and understand the rate capability of the NiCo_2_O_4_/rGO/NiO composite electrode, the charge/discharge measurements were performed. Figure 5b gives the discharge curves of the NiCo_2_O_4_/rGO/NiO electrode at various current densities. The corresponding specific capacitance was calculated to be 2644 mF cm^−2^ at a low current density (1 mA cm^−2^), and 1821.6 mF cm^−2^ at a high current density (100 mA cm^−2^).

**Fig. 6:**
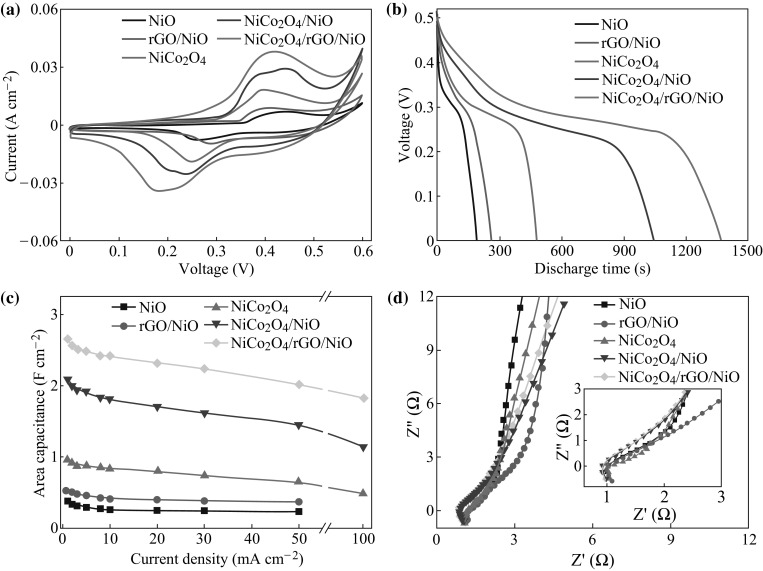
Electrochemical properties of the samples: **a** CV curves at 5 mV s^−1^, **b** GCD curves at 1 mA cm^−2^, **c** the corresponding specific capacitance, and **d** EIS plots of the samples (*inset* is the enlarged plot of the high-frequency regions). (Color figure online)

In order to confirm the outstanding electrochemical performance of the sandwich-like NiCo_2_O_4_/rGO/NiO heterostructure composite, the electrochemical properties of the NiO, rGO/NiO, NiCo_2_O_4_, and NiO/NiCo_2_O_4_ were also tested. For comparison, CV curves of the NiO, rGO/NiO, NiCo_2_O_4_, NiCo_2_O_4/_NiO, and NiCo_2_O_4_/rGO/NiO at a scan rate of 5 mV s^−1^ are illustrated in Fig. 6a. Clearly, the enclosed area of the sandwich-like NiCo_2_O_4_/rGO/NiO heterostructure material is much larger than those of other samples, indicating that the NiCo_2_O_4_/rGO/NiO has a lager areal capacitance. Figure 6b shows the discharge capacitance of NiO, rGO/NiO, NiCo_2_O_4_, NiCo_2_O_4_/NiO, and NiCo_2_O_4_/rGO/NiO at a current density of 1 A cm^−2^. Similarly, the sandwich-like NiCo_2_O_4_/rGO/NiO heterostructure material delivers a higher specific capacitance than others.

Figure 6c illustrates specific capacitance as a function of current density of NiO, rGO/NiO, NiCo_2_O_4_, NiCo_2_O_4_/NiO, and NiCo_2_O_4_/rGO/NiO. Within the current density range of 1–100 mA cm^−2^, the area-specific capacitance of the samples has the order of NiO < rGO/NiO < NiCo_2_O_4_ < NiCo_2_O_4_/NiO < NiCo_2_O_4_/rGO/NiO. Since the area-specific capacitances of NiO and rGO/NiO are too small at a high current density of 100 mA cm^−2^, the data are not presented in Fig. 6c. The area-specific capacitances of the NiO, rGO/NiO, NiCo_2_O_4_, NiCo_2_O_4_/NiO, and NiCo_2_O_4_/rGO/NiO were 374, 516.8, 975.5, 2092, and 2644 mF cm^−2^ at a low current density (1 mA cm^−2^), respectively.

In general, EIS is usually used to investigate the performance of electrochemical capacitors, such as internal resistance and capacity [18, 19]. The EIS data were commonly analyzed by using Nyquist plots, in which the frequency response of the electrode/electrolyte system and the plots of the imaginary component (*Z*″) of the impedance against the real component (*Z*′) are presented [29]. As shown in Fig. 6d, the EIS curves exhibit similar forms with inconspicuous semicircle in the high-frequency region and an almost straight sloping line in the low-frequency region. The inconspicuous semicircle region indicates low faradaic resistance of the materials and good electrical conductivity between the samples and NF [30, 31].

**Fig. 7:**
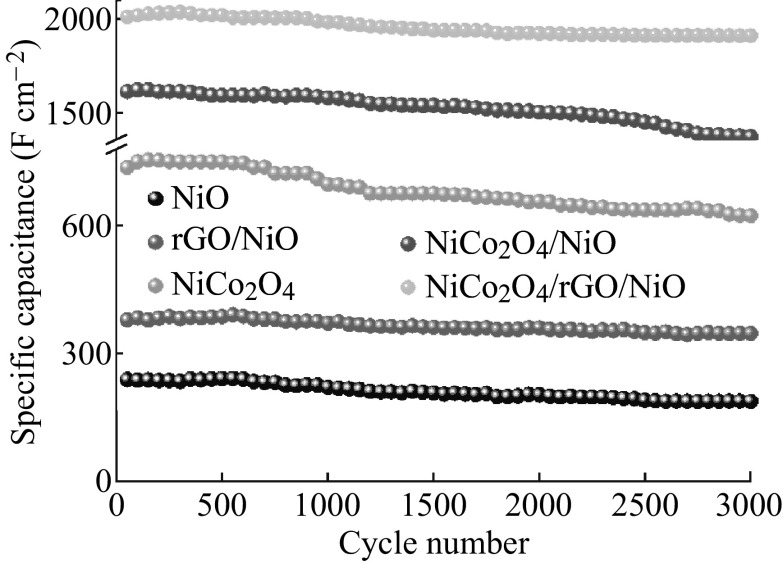
Cycle performance of the NF, NiO, rGO/NiO, NiCo_2_O_4_, NiCo_2_O_4_/NiO, and NiCo_2_O_4_/rGO/NiO at current density of 30 mA cm^−2^. (Color figure online)

Cycling stability is another critical factor in evaluating the electrochemical properties of supercapacitors. The cycling stability of the sandwich-like NiCo_2_O_4_/rGO/NiO heterostructure was evaluated by the repeated GCD measurement at a current density of 30 mA cm^−2^, as shown in Fig. 7. Obviously, the specific capacitance of the NiCo_2_O_4_/rGO/NiO composite slightly decreases to 97.5% for the first cycle after 3000 time’s tests, demonstrating the excellent cycling stability.

**Fig. 8:**
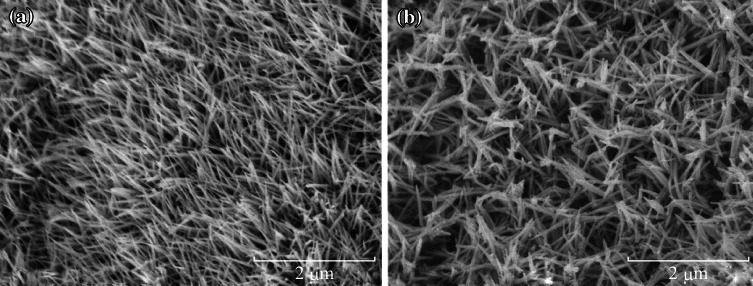
SEM images of the NiCo_2_O_4_/rGO/NiO heterostructures: **a** before, and **b** after cycle test

The morphology of the sandwich-like NiCo_2_O_4_/rGO/NiO heterostructure after cycling test was further investigated (see Fig. 8).Obviously, the morphology and structure of the NiCo_2_O_4_/rGO/NiO are well-preserved even after 3000 time’s cycling. As shown in Fig. 8b, the NiCo_2_O_4_ nanoneedles could be seen obviously even after 3000 time’s charge–discharge test. However, compared with the initial morphology (shown in Fig. 8a), some tips of the NiCo_2_O_4_ nanoneedles are broken during the charge–discharge process. This may reduce the specific capacitance.

All the above results confirm that the sandwich-like NiCo_2_O_4_/rGO/NiO heterostructure can improve the electrochemical performance for supercapacitor application. The possible mechanism may be ascribed as follows: (i) NiCo_2_O_4_ is a kind of p-type semiconductor material. If it is coupled with other semiconductors, it will introduce impurity band effect which can greatly enhance the electrical conductivity as electrode materials [32–34]. The unique structure characteristics of the NiCo_2_O_4_/rGO/NiO composite present a promising candidate for high-performance supercapacitors electrode materials. (ii) The 2D NiO nanoplates, 1D NiCo_2_O_4_ nanoneedles, and 2D rGO film constructed to 3D porous structure on NF, allow completely exposing to the electrolyte, and thus optimize the electrochemical accessibility [26, 27]. (iii) The hierarchical architecture which directly grew from the current collector avoids the use of binders and substantially reduces the dead volume in the electrode, and therefore would be favorable for the migration of hydrated ions in the electrolyte to the surface of the electrode [15, 35]. (iv) The existence of graphene enhances the conductivity, and therefore enhances the specific capacitance. Also the flexible graphene between NiO and NiCo_2_O_4_ could buffer against the local volume change during the charge–discharge process, as well as alleviate the pulverization and aggregation of the electrode material [36–38]. (v) Moreover, the synergistic effect of each component would lead to fast ion/electron transfer and enhance flexibility, which finally results in the improvement of electrochemical performance.

## Conclusions

The sandwich-like NiCo_2_O_4_/rGO/NiO heterostructure composites with high electrochemical performance were synthesized on NF directly via a three-step hydrothermal strategy. The as-fabricated heterostructure composites exhibit excellent electrochemical performance including high specific capacitance, good electrochemical stability, and excellent rate capability. Its remarkably enhanced electrochemical performance is attributed to the unique hierarchical sandwich-like structure and the synergistic effects among NiCo_2_O_4_, rGO, and NiO. The results suggest that the sandwich-like NiCo_2_O_4_/rGO/NiO heterostructure composites will be promising electrode materials for high-performance sustainable energy storage devices.
